# Estimating Extracellular Spike Waveforms from CA1 Pyramidal Cells with Multichannel Electrodes

**DOI:** 10.1371/journal.pone.0082141

**Published:** 2013-12-31

**Authors:** Sturla Molden, Olve Moldestad, Johan F. Storm

**Affiliations:** Department of Physiology, Insitute of Basic Medical Sciences, University of Oslo, Oslo, Norway; Consejo Superior de Investigaciones Cientificas - Instituto Cajal, Spain

## Abstract

Extracellular (EC) recordings of action potentials from the intact brain are embedded in background voltage fluctuations known as the “local field potential” (LFP). In order to use EC spike recordings for studying biophysical properties of neurons, the spike waveforms must be separated from the LFP. Linear low-pass and high-pass filters are usually insufficient to separate spike waveforms from LFP, because they have overlapping frequency bands. Broad-band recordings of LFP and spikes were obtained with a 16-channel laminar electrode array (silicone probe). We developed an algorithm whereby local LFP signals from spike-containing channel were modeled using locally weighted polynomial regression analysis of adjoining channels without spikes. The modeled LFP signal was subtracted from the recording to estimate the embedded spike waveforms. We tested the method both on defined spike waveforms added to LFP recordings, and on in vivo-recorded extracellular spikes from hippocampal CA1 pyramidal cells in anaesthetized mice. We show that the algorithm can correctly extract the spike waveforms embedded in the LFP. In contrast, traditional high-pass filters failed to recover correct spike shapes, albeit produceing smaller standard errors. We found that high-pass RC or 2-pole Butterworth filters with cut-off frequencies below 12.5 Hz, are required to retrieve waveforms comparable to our method. The method was also compared to spike-triggered averages of the broad-band signal, and yielded waveforms with smaller standard errors and less distortion before and after the spike.

## Introduction

Extracellular (EC) recordings of action potentials yield “spikes” from a population of neurons in the vicinity of the recording electrode. These spikes are embedded in background voltage fluctuations known as the “local field potential” (LFP). In order to study the function of individual neurons with this method, the spikes must be extracted from the LFP and separated into groups of spikes originating from the same neuron. This separtation requires unsupervised pattern classification (data clustering), which in this context is often called “spike sorting” [1], [2]. Extracting the spikes from the LFP have been given less attention than spike sorting [2], [3], perhaps because analog or digital filters [4]–[6] with a cut-off frequency at a few hundred Hertz have been implicitly trusted to do the spike and LFP separation correctly [2], [3]. In this paper we will show that the fundamental assumption that “spikes” and LFP are spectrally distinct cannot generally be made.

Extracellular (EC) recordings of action potentials from single neurons (unit recordings) have been widely used for examining neural activity and coding, starting almost a century ago with recordings of spikes in single nerve fibers with a Lippmann electrometer [7], [8]. This method has been immensely useful for studying neural activity and coding in a wide range of species and neural structures, ranging from single nerve fibers in invertebrate sensory organs [9], [10] to cortical neurons involved in cognition, memory, and navigation in awake, behaving animals and humans [11]–[19]. However, most such studies so far have used unit recording only for determining spike patterns, firing frequencies, and spike timing [20], so the detailed waveform of each EC spike has normally not been of interest, except spike sorting [21]–[23] and sometimes differentiating cell types [24]. Studies of the EC waveform have also not considered the effects of routine filtering [25] or considered cut-off frequencies below 300 Hz [3].

Nevertheless, the waveform of the EC spikes contains information about the biophysical mechanisms underlying the action potentials [26], and may thus be used to reveal membrane properties of the recorded neurons. In order to utilize this information, one must be able to faithfully extract the EC spike waveform by separating it from background activity of surrounding cells and other irrelevant signals. Recordings of EC spikes in the intact brain are nearly always embedded in background field potential fluctuations known as the LFP, which are difficult to reliably separate from the spike waveforms.

**Figure 1:**
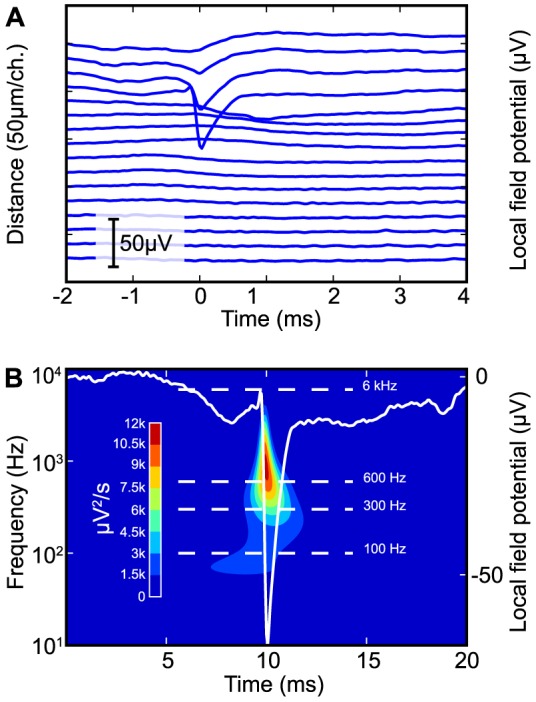
Depth profile and wavelet spectrum of the spike triggered hippocampal LFP. (**A**) Depth profile of the local field potential (LFP) in the mouse hippocampus, subfields CA1 and DG. The plotted signals are average LFP traces triggered on the action potentials from an isolated CA1 pyramidal cell. The LFP was recorded using a 16-site laminar electrode array, with 50 µm between the recording sites. The LFP traces are broad-band filtered between 0.5 Hz and 6 kHz. (**B**) Wavelet spectrum (Paul mother wavelet, m = 4) of the fourth LFP trace in panel A, counting from the top, *i.e.* the one with the strongest spike amplitude in the CA1 LFP. The relative signal power is color coded from red (highest) to blue (no signal). Common high-pass filtering frequencies used for extracellular multi-unit recording (300 Hz and 600 Hz) intersects the bulk of the wavelet spectrum. The waveform corresponding to the wavelet spectrum is shown in white. The Paul wavelet was chosen because its asymmetric shape and high temporal resolution and makes it well suited for analyzing spike waveforms.

Traditional linear filters are often insufficient because the frequency bands of the LFP and the EC spike waveforms are overlapping. Figure 1 shows an average extracellular spike waveform from the mouse hippocampus, its depth profile from CA1 (1A, upper trace) to the dentate gyrus (1A, bottom trace), and its wavelet spectrogram (1B; CWT, Paul wavelet basis, 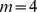). The spikes were recorded from a mouse CA1 pyramidal cell during urethane anesthesia. The lower range of the frequency spectrum is below 100 Hz, similar to what has previously been reported [27]. The highest frequency oscillatory components in the hippocampal LFP can reach 300 Hz [28]. A 600 Hz cut-off frequency, commonly used for extracellular recording [29], will clearly intersect the wavelet spectrum (Figure 1B). Filtering at frequencies above 100 Hz is therefore expected to interfere with the EC spike signal we wish to isolate. Only the fast components of the EC spike will be retained, while the slower components that are typically produced by slowly activating or small ionic currents, including after-hyperpolarizations (AHPs) and after-depolarizations, will be repressed [30]–[33]. In this paper we will show that filtering frequencies as low as 12.5 Hz are needed to preserve the waveform.

**Figure 2:**
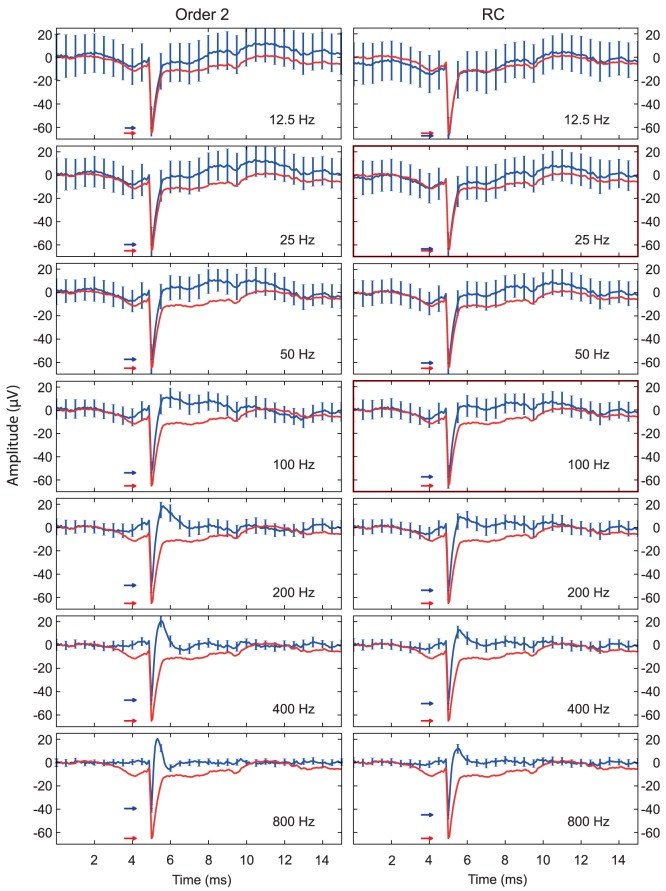
High-pass filtering an average CA1 spike waveform. The figure shows the effect of passing the spike waveform from Figure 1B through various high-pass filters, after inserting it into the recorded hippocampal local field potential. The red trace indicates the inserted waveform (and thus the desired output), the blue trace indicates the average of the filtered signal with standard deviation in the error bars. High-pass filtering frequencies increase from 12.5 Hz (top) to 800 Hz (bottom). An order-2 Butterworth filter is used for the left column, a single-pole (RC) filter is used for the right column. The filter-types were chosen because they correspond to common filter designs used for extracellular multi-unit recording. The gradual development of the waveform distortion is evident from the upper to the lower panels, with significant distortion already ad 12.5 Hz for order-2 Butterworth and 12 Hz for RC. The waveform distortion includes narrowing of the spike, depression of the amplitude, and positive over-shoot after the spike. The red and blue arrows indicate the peak amplitude of the inserted signal (red traces) and the average filtered signals (blue traces). The distance between the blue and red arrow indicate the bias in the amplitude estimate. The development of the amplitude bias in the left column is also shown in greater detail by the dotted lines in Figure 5A–E.

In addition to spectral overlap between LFP and the EC spike, high-pass filters will also distort the shape of the fast components in the waveform. There are two sources for these artifacts. First, all analog infinite impulse-response (IIR) high-pass filters (including Bessel) will produce an asymmetric distortion of the waveform, resulting from a so-called “non-linear phase response” [4]–[6]. Second, some high-pass filters will cause overshoots in the signal, thus introducing “ringing” in the waveform. Figure 2 shows the effect of applying an order-2 Butterworth high-pass filter or a single-pole (RC) high-pass filter with cut-off frequencies from 12.5 to 800 Hz. An analog version of the order-2 Butterworth filter design with 600 Hz cut-off is often used to isolate single units in extracellular recordings [21], [22], [24], [29], [34]. The output signals (blue trace in Figure 2) from cut-off frequencies in the range 200–800 Hz are severely distorted. The slow components are erased, the peak is narrowed, and the peak amplitude is reduced (arrows in Figure 2). For cut-off frequencies below 100 Hz, the distortion of the slow components mainly affects the latter half of the spike waveform. In Figure S1 we have tried to use the same filters with zero-phase “forward-backward” filtering. Here the distortion of the slow components affects the spike waveform equally in both ends, showing that the asymmetry in Figure 2 is indeed produced by the non-linear phase response of the filters we have used. Taken together, Figures 2 and S1 suggests that high-pass filters with cut-offs as low as 12.5 Hz is needed to properly preserve the spike waveform. This is far below the conventional filter settings used for extracellular spike recording. When considering the common choices of filtering frequencies, such as above 200 Hz in Figures 2 and S1, an EC spike waveform with this amount of filter distortion is obviously not suited for studying ion channel functions or any other biophysical properties underlying the action potential.

In addition to ion channel function, waveform distortion might also have implications for the success-rate in waveform clustering (often called “spike sorting”) [2], [3], [21]–[23]. As extracellular spikes originate from action potentials in a local population of neurons (“multiple-unit recording”), isolation of action potentials from single neurons (“single-units”) is a blind data classification problem requiring cluster analysis. During the process of clustering, each spike is assigned to a unit (or hypothetical cell) on the basis of extracted waveform features. Distortion of the EC spike waveform can therefore cause the spike sorting process to be affected by spurious waveform features. Distortion might also mask waveform differences that might have guided correct separation of action potentials from two different cells. Digital filtering might therefore increase the incidence of both “type-1” (false negative) and “type-2” (false positive) classification errors.

Filter-free methods might also be used to recover the extracellular spike from the background LFP. The simplest approach is perhaps to compute a spike-triggered average of the broad-band signal, which requires a sufficient number of spikes recorded under nearly identical conditions and unambiguous vertical alignment. However, this method can only provide the average waveform, and is susceptible to systematic errors from phase-locking with the LFP (an example of which we will demonstrate in this paper). Another approach is to record the LFP and subtract it with an operational amplifier [6]. However, the LFP will seldom be exactly the same when recorded from different sites in the brain. A better option might be to use a weighted sum of LFP recorded from different, close-by reference sites to subtract the LFP [35]–[37]. For example, arrays of micro-electrodes allow simultaneous recording of LFP from positions in close proximity [38]. Such “array processing” has previously been used for various electrode arrays for removing high-frequency noise from extracellular recording [39]–[41], but not, to our knowledge, for separating background broad-band LFP from extracellular spikes.

In this paper we show that recordings from a laminar electrode array can be used to recover the waveform of EC action potentials of hippocampal pyramidal cells from spike recordings embedded in LFP, without using frequency selective filters. The method is compared with traditional high-pass filtering and spike-triggered averages of the broad-band signal.

## Results

### Modelling the background LFP

**Figure 3:**
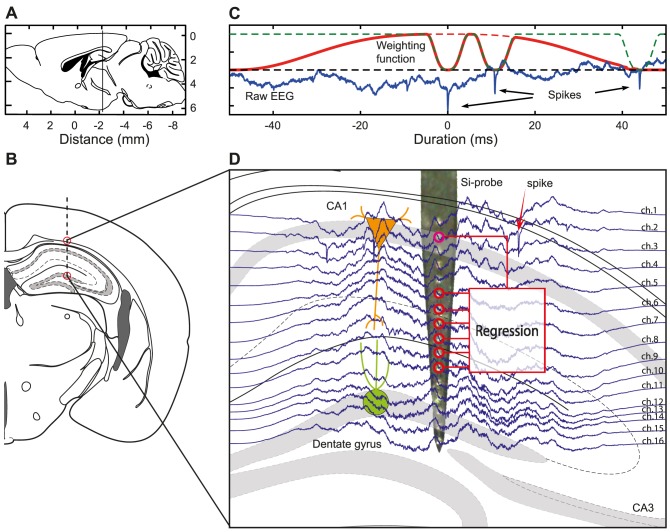
Principle for modelling and subtracting the LFP. (**A**) Rostrocaudal localization of the laminar electrode array used to record the local field potential (LFP) in the mouse hippocampus. (**B**) Localization of the laminar electrode array in the coronal plane of the mouse brain (lateromedial and dorsoventral localization). (**C**) The panel shows the time windows used to model the LFP. The dashed red line is a tricube window (here: 100 ms wide) used to get a local estimate around the spike in the center (0 ms). The dashed green line is flipped tricube windows used to remove the influence of the spike waveform from the fitted LFP model. Three spikes are edited out. The solid red line is the weight function actually used to estimate the regression model, computed as the product of the dashed red and green lines. The corresponding LFP signal is shown in blue. The dashed black line is the DC level. (**D**) Depth profile of temporally weighted LFP traces recorded from the mouse hippocampus. A microscope image of the tip of the laminar electrode array is shown in the background. The 16 light spots in the center of the probe are the recording sites. The electrode array can be seen to cover the full depth profile trough the hippocampal layers from CA1 to DG. Recording from a set of reference channels in a laminar electrode can give an estimate of the local field potential on a different channel with spikes, thus allowing the estimated LFP to be subtracted in order to recover the spike waveforms from the LFP. The recovered waveforms are expressed as the prediction errors. The illustrations of the mouse brain (**A**, **B** and **D**) are adapted from Paxinos and Franklin [56].

A number of methods for multi-reference noise cancellation [35] in LFP or EEG recording have been described previously. These include linear regression, principal components analysis, wavelet transforms, and artificial neural networks [36], [39]–[41]. The general principle is to model the common “noise” from a set of reference electrodes, and subtract the estimated noise from the channel of interest. The recovered signal is the difference between the recording and the predicted noise. Here we develop a similar approach for isolating single-unit spikes from broad-band LFP data, and test the method on extracellular recordings from pyramidal cells in the hippocampus of anesthetized mice (Figure 3).

In the rodent hippocampus, cell bodies of principal cells in CA1 and dentate gyrus are organized in dense layers less than 100 µm thick (Figure 3A–B). When recording from positions more than 150 µm outside the soma layers, e.g. in the *stratum radiatum*, action potentials from the principal cells will normally not be visible on the recording. As illustrated in Figure 3D, a 16-channel laminar electrode array (50 µm spacing) can simultaneously record from almost all layers of the mouse hippocampus, *i.e.* from the *stratum pyramidale* of CA1 to the hilus of the Dentate gyrus (Figure 3D). Recordings from the *stratum radiatum*, which normally do not contain spikes, might therefore be used to track the perisomatic LFP with no or minimal influence from action potentials. In order to model the perisomatic LFP from recording with a laminar electrode array, we used multiple regression [42]. The chosen regressors were a set of channels without spikes, recorded from the *stratum radiatum* below the CA1 pyramidal layer.

The background LFP in the hippocampus cannot be assumed to be stationary since it contains transient events (sharp waves, dentate spikes), multiple oscillatory states (e.g. REM sleep and slow-wave sleep), and oscillations with fluctuating frequency and amplitude, such as theta and gamma oscillations [13], [43], [44]. The model must be able to adapt to these changes in the LFP. This is possible because the LFP changes on a much slower time-scale than the duration of an EC action potential.

The LFP can be modeled as a non-linear function of both the radial distance to the neuron and the geometry of the cell and transmembrane currents in different compartments (Gold *et al.* Equation 3 [26]). A regression model used to track the perisomatic LFP should therefore be able to approximate non-linear dependencies between the LFP signals recorded at different locations. We used a cubic polynomial model, and included interaction terms to account for cross-talk between different channels in the electrode array [41].

To localize the transient spike events in the LFP, we first band-pass filtered the perisomatic LFP between 600 Hz and 6 kHz and interpolated the signal to 96 kHz. Spikes in the 96 kHz trace were then triggered on the basis of their amplitude (manually adjusted trigger level). The non-stationary nature of the LFP was modeled using local time windows. These temporal changes originate from shifting EEG states and transient spikes events in the LFP. To avoid having the prediction model influenced by the extracellular APs, 10 ms time windows surrounding each triggered spike (tentative extracellular AP) was down-weighted using a modified tricube function. We then selected a much broader time-window (e.g. 200 ms) around each spike to fit a local regression model. The duration of this window was chosen to be much broader than an extracellular action potential, yet short enough for the EEG state to be considered approximately constant. In our experience, the outcome of the method is relatively insensitive to the exact choice of kernel (see Figure S3). The function used to generate the local time-windows is described in Equation 1, and Equation 2 describes the final weight functions.
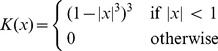
(1)

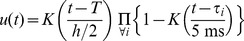
(2)


Equation 2 gives the temporal weights 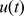 around the waveform detected at time 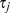, which are used to model the LFP in the vicinity of time 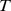. Here, 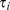 is the time-stamps of all detected spikes in the whole recording and 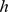 is the duration of the time window, e.g. 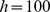 ms. The whole period 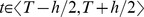 was used to model the LFP in the vicinity or time-stamp 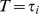 (Figure 3C).

Multiple regression model was used to predict the pyramidal layer LFP in vicinity of the detected spikes, with recorded signals from other hippocampal layers as input (Figure 3D).
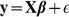
(3)Here 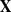 is a matrix or regressors. The columns in X consist of a constant term, the signals from the reference channel, the signals from the reference channels squared, the signals from the reference channels cubed, and pairwise interaction terms between the reference signals. Higher order interaction terms were not included. 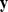 is a column vector describing the perisomatic LFP. All the signals were standardized to their 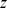-score (zero mean and unit variance) before being used as regressor or response. The regression model was fitted using general least squares assuming a diagonal noise covariance matrix, for which the ML estimate is found by solving
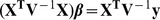
(4)for 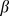. We assumed the error covariance matrix V has diagonal elements 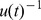 and 0 off-diagonal elements. This is equivalent to weighting each sample by 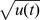 [42]. The general least-squares estimate was computed using QR factorization [45].

### Computational results

Figure 1 shows the average extracellular AP from a CA1 pyramidal cell. The depth profile throughout the hippocampal layers is shown in panel A and the wavelet spectrogram is shown in panel B. It is evident from the figure that the frequency spectrum reaches down to 100 Hz or below. Figure 2 shows the effect of passing the spike waveform through two common high-pass filters at various cut-off frequencies. It illustrates that the order-2 Butterworth distorts the spike waveform even at 12.5 Hz, particularly at its rear end. The distortion seems to first affect the rear part of the waveform, and then gradually change the main spike waveform, leading to narrowing and depression of its amplitude (arrows in Figure 2). A cross increasing cut-off frequency, the filtered waveform gradually acquired a positive overshoot which was not present in the unfiltered spike. This should not be mistaken for extracellular signature of the AHP or any real biophysical phenomenon, as it is generated by the impulse response of the filter. The RC filter has similar problems as the 2-pole Butterworth, but the waveform distortion is not so pronounced and develops more slowly. Offline (zero-phase) filters also have this behavior (Figure S1).

**Figure 4:**
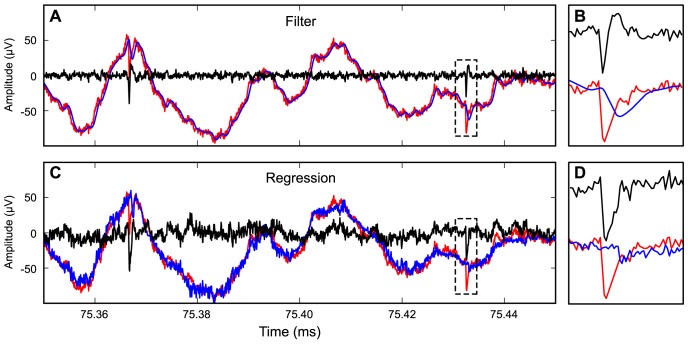
LFP predictions from linear filters and multiple regression. (**A**) Prediction of LFP (blue) and spikes (black) using order-2 Butterworth filters at 600 Hz. The original broad-band signal is shown in red. The distortion of the spike waveform can be seen in the expanded traces in B. (**B**) Example of an extracellular spike in the original signal (red) and the corresponding high-pass (black) and low-pass (blue) filtered traces using an order-2 Butterworth filter. (**C**) Prediction of LFP and spikes using linear regression, with a sliding time-window of 100 ms. There is higher noise in the LFP estimate (blue in B) and the spike estimate (black in B) than in the original broad-band signal (red in A) and the filtered spike trace (black in A). (**D**) The same spike with original broad-band signal (red) residual from multiple regression (black) and LFP prediction from multiple regression (blue).

**Figure 5:**
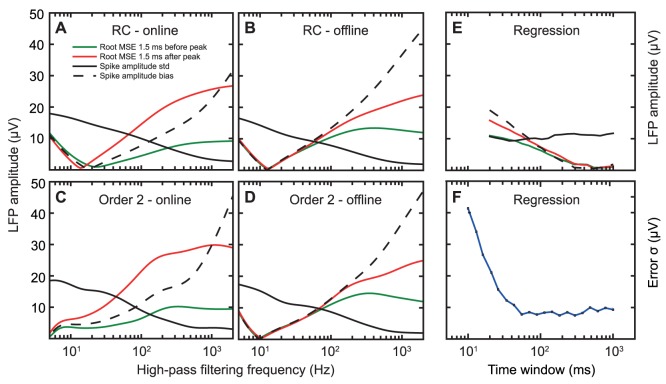
Effect of filtering frequency and time window size on the prediction error and variability. (**A–D**) Quantification of the frequency dependent spike-waveform distortion from an order-2 Butterworth high-pass filter. The red and green lines show the root mean square (RMS) errors in the 1.5 ms before and after the spike. The solid black line shows the standard deviation of the filtered amplitude at the peak of the spike. The dashed black line shows the bias (absolute error) in the spike amplitude. (**E**) The effect of time window size on the waveform distortion from the regression estimator. (**F**) The effect of time window size on the variability in the LFP regression estimate. The standard deviation is smallest around 100 ms, which corresponds to half the period of a theta cycle (5 Hz) in the hippocampal LFP under these recording conditions (urethane anesthesia).

Using recordings from laminar electrodes we correctly recovered extracellular spike waveforms embedded in LFP (EEG), without introducing filter artifacts. Figure 4 shows an example of LFP predictions with linear filtering (A–B) and multiple regression (C–D). The upper trace (Figure 4A) was computed using an order-2 Butterworth filter (600 Hz cut-off). The low-pass filtered trace (blue) seems smooth, and there is little noise in the high-pass filtered trace (black), making two spikes obvious. However, by careful inspection of Figure 4B we can see that the shape of the spike is distorted. The filtered EC action potential is narrower, and the waveform has acquired a trailing “AHP-like” bump (Figure 4B). Figure 2 shows how this distortion develops as a function of filtering frequency. Figure 5A–D try to quantify how the variability and distortion develops with increasing filtering frequency. The green line shows the root mean square (RMS) error in the 1.5 ms period before the peak of the spike, the red line shows the RMS error in the 1.5 ms period after the peak of the spike. The figure shows that the filtering frequency has to be well below 100 Hz, and even below 10 Hz, for the errors and spike amplitude bias to be small. That is, the filter must only remove the slowest components of the hippocampal LFP, such as theta and delta waves. Here, the theta waves are at 4 Hz due to urethane, which incidentally corresponds to the minimum-error filter settings (Figure 5A–D). In contrast, the regression estimate obtained by our new method has higher noise (Figure 4C), but the shape of the spike does not have the extra trailing bump, and is not narrower than the spike in the raw signal (Figure 4D). The error in the regression estimate was dependent on the time window (Figure 5E and F and Figure S3). As can be seen in Figure 5F, the error reaches a minimum around 50–400 ms. This is probably related to the fact that 200 ms corresponds roughly to one period of the hippocampal theta when recording from a urethane-anaesthetized mouse (Figure 4), as used in these experiments.

**Figure 6:**
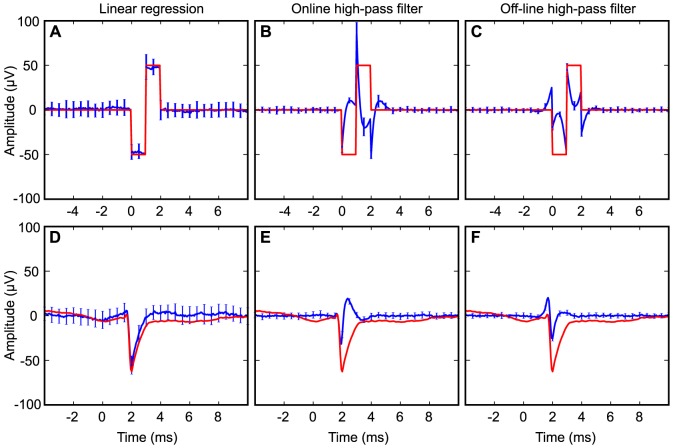
Waveform estimation with high-pass filtering and multiple regression. (**A–C**) Estimation of a biphasic square-wave pulse using (A) multiple regression, (B) on-line high-pass filter, or (C) anti-causal high-pass filter. The red line corresponds to a dummy signal inserted into the hippocampal LFP. The blue line shows the average regression or filter estimates with standard deviations in the error bars. (**D–F**) Estimation of an extracellular spike with the same methods as in A–F. The full depth profile from Figure 1A was inserted into the hippocampal LFP.

When testing with inserted dummy waveforms, regression analysis was able to correctly extract the shape of an extracellular AP, as well a biphasic square-wave pulse (Figure 6A). In contrast, the waveforms obtained after high-pass filtering (order-2 Butterworth, 600 Hz; blue traces in Figure 6, B–C and E–F) are very different from the input signals (red traces in Figure 6, B–C and E–F). The regression estimates (blue traces in Figure 6A and D) are clearly more similar to the input signals (red traces). Filtering off-line also allows the use of linear-phase, by applying the filter in both forward and reverse directions. While this anti-causal filtering gives a symmetric impulse response, it does not help much to make the filtered output closer to the original. Figure 6F shows that applying the filter in reverse can actually turn a rather symmetric spike waveform (red trace) into an asymmetric one after filtering (blue trace). This is rather paradoxical as the purpose of a linear-phase filtering is to preserve the symmetry of a symmetric waveform. Given that windowed sinc “FIR” (finite impulse-respone) filters also have linear phase, this popular digital filter design might not be better than recursive IIR filters for spike waveform isolation [5].

**Figure 7:**
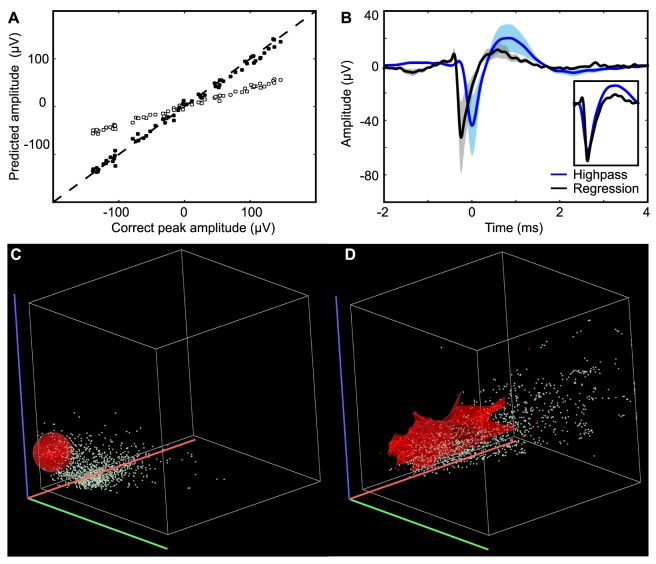
Amplitude distribution and clustering-cutting scatter-plots. (**A**) Predicted amplitudes of EC spikes with variable amplitudes using high-pass filtering (open circles) or multiple regression (filled dots). The dashed diagonal indicates perfect estimate. The amplitude estimated from high-pass filtering is negatively biased, whereas regression produces an unbiased estimate. (**B**) Average waveforms with standard deviation from the cell isolated in A and B. It can be seen how high-pass filtering delays the waveform, depresses the amplitude, and adds a trailing “bump”. (**C–D**) Scatter-plot of the amplitudes of recorded EC spikes from CA1 cells on three channels (range 0–350 µV) on a laminar electrode using high-pass filtering (C) and multiple regression (D). The shape of the cluster of amplitudes produced by an isolated cell (red dots) is indicated with a transparent red surface. It can be seen how high-pass filtering and multiple regression produce different ranges and correlation structures for spike amplitudes of the cell.

In addition to distorting the spike shape, the traditional high-pass filtering also introduced a systematic error in spike-amplitude estimation (Figure 2, 5A–D and Figure 7A). During an epoch of recorded theta, 63 spikes with random amplitudes between 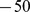 and 150 µV were inserted at the trough of the LFP. With the high-pass filter, the peak amplitude of the spike was systematically underestimated, but was still proportional to the correct amplitude. In contrast, linear regression gave a more correct estimate of the peak amplitude (Figure 7A). The variability in the waveform estimates (mean and standard deviation) with regression and high-pass filtering is shown in Figure 7B.

Multichannel spike recordings allow spike-sorting based on spike amplitudes, since the latter depend on the distances between the cell and the electrodes [2], [22]–[24], [26]. The peak amplitudes of the high-pass filtered spikes were proportional to the correct spike amplitude, which indicates that they might still be used for spike-sorting. The amplitudes of the spike-waveforms formed clusters with both high-pass filtering and multiple regression, but the shape of the clusters in the amplitude scatter-plots were very different (Figure 7C and 7D). The possible consequences of the different cluster shapes for classification errors in spike-sorting [2], [3], [23] remain to be investigated.

**Figure 8:**
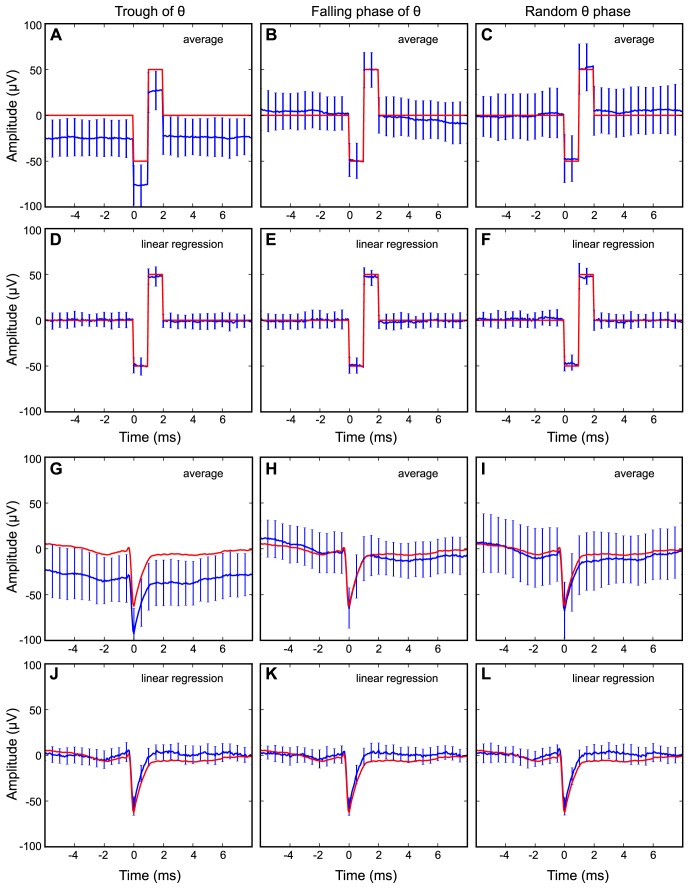
Waveform estimation with spike-triggered average and multiple regression. (**A–F**) Estimation of a biphasic square-wave pulse phase-locked to different theta phases with spike-triggered average (A–C) and multiple regression (D–F). The red line corresponds to a dummy signal inserted into the hippocampal LFP. The blue line shows the average regression or filter estimates with standard deviations in the error bars. (**G–L**) Estimation of an extracellular spike phase-locked to different theta phases with spike-triggered average (G–I) and multiple regression (J–L). The full depth profile from Figure 1A was inserted into the hippocampal LFP. Spike-triggered average produces larger standard deviation and phase-dependent systematic error.

Another procedure that has been used to estimate spike waveforms is averaging of extracellular traces (see *e.g.* [25], [37]). However, this procedure also typically leads to larger variability (Figure 8A–C and G–I) than regression (Figure 8D–F and J–L). For example, averaging of EC traces from the hippocampus can cause systematic errors because the phase of the EC theta oscillation (LFP) affects the average. Thus, near both the peak and the trough of the hippocampal theta oscillations, the averaged trace showed an apparent DC-offset (Figure 8A and G; only recordings at the theta trough are shown, but the effect on spikes near the peak was very similar). Furthermore, on the falling or raising slopes of the EC theta oscillation, the baseline appears to have a sloping trend (Figure 8B and G; only falling phase shown). This is important to keep in mind since many neurons are phase modulated with respect to the LFP oscillation. In contrast, the regression estimate showed smaller error and no dependency on the phase of the LFP theta oscillation (Figure 8D–F and J–L). Apart from these differences in baseline, a broad-band average was able to isolate the correct shape of the spike waveform.

## Discussion

We have shown that simultaneous recording from a multi-channel laminar electrode array can be used to recover the waveform of pyramidal cell extracellular spikes from the background LFP, without using frequency selective filters. We compared the method with traditional high-pass filtering and spike-triggered averaging of the broad-band signal, and demonstrated that multiple regression can recover the waveform of extracellular spikes with less artifacts and distortion than high-pass filtering. Of course, the correction based on LFPs recorded at nearby channels can never be perfect, but any error caused by variable local differences between LFPs are generally very small compared to the EC spike amplitude. Thus, we found that the slight distortions introduced by the multiple regression method proved to be far smaller than the distortions introduced by high-pass filtering, in all the examples we tested.

### Waveform estimation with multiple regression

Testing with inserted waveforms showed that weighted multiple regression (Equation 1–4, Figure 3) could recover the waveform of individual spikes from the field potential (Figures 4, 5 and 7), but this method has some limitations. First, it requires that the field potential is recorded with a sufficient number of reference electrodes that are “close enough” to the spike-recording electrode(s) to record essentially the same background LFP, but still does not record action potentials to any significant degree. Although reference electrodes are located in neuropil or other structures sufficiently far from neuronal somata, large axons or dendrites might still produce strong extracellular action potentials. Optimal positioning of reference electrodes is therefore paramount to the effectively of this method. Second, if an estimate of the LFP between spikes is required (as in Figure 4C), a sliding time-window might not yet be computationally tractable. The higher variance in the multiple regression estimator produced clusters with a larger range of amplitudes than high-pass filtering, and this might have consequences for spike-sorting errors (Figure 7C and 7D). Higher variance is expected to produce larger rates of random classification errors; lower waveform distortion is expected to produce smaller systematic classification errors. The exact consequences of filter methods on spike-sorting errors remain to be investigated. For spike detection and time-stamping, using a filter-based approach will be more efficient due to the smaller computational demand. Also, recovering the waveforms is not necessary for unit recording analysis, which only focuses on spike-timing and the temporal structure of the firing patterns [4], [20], [37]. In contrast, recovering the correct waveform of the extracellular spike is required for any more detailed biophysical investigation, including computing the current-source density of single action potentials [46].

It is particularly important to use a numerical model that can approximate a non-linear relationship between LFPs on the recording sites. The LFP generated by a linear current source is a non-linear function of the radial distance to the source. A compartmental model of a neuron will produce one linear current source for each dendritic and axonal compartment, as well as a point source for the soma, see *e.g.* Gold *et al.* Equation 3 [26]). To approximate this non-linearity, we used a cubic polynomial model, and included interaction terms to account for cross-talk between the different channels in the multi-channel electrode array [41]. Other estimation procedures can be used to model the non-linear relationships in the LFP between the channels as well. One example is a feed-forward multilayer artificial neural network with sigmoid activation function [47].

Artificial neural networks have previously been used for EEG prediction for the purpose of detecting aberrant activity patterns in human scalp EEG, *e.g.* epileptiform spikes [36]. The method is conceptually similar to the regression analysis we used in this paper, but the authors did not describe how the waveform shapes were preserved, and it was tested on a different kind of data (scalp EEG). Their method also used recursive estimation [35] to continuously process the EEG data. Our procedure only aimed to predict the EEG in vicinity of detected spikes.

Decorrelation, principal components, and multiple regression has also been used for cancellation of spatially correlated high-frequency noise in extracellular tetrode recordings [39], [41], as have wavelet transforms [40]. However, these reports have focused on denoising the high-frequency band of the LFP, and not taken the correctness of the waveform shape into account. The observation that it is particularly efficient to use multi-channel denoising for removing the common noise in the high-frequency band suggests that it might be better to decompose the LFP with a filter-bank that permits perfect reconstruction (e.g. a wavelet transform), and predict each LFP sub-bands with independent regression models [40], [48]). Experience from image processing also suggests that random noise is mainly present in the high-frequency “detail coefficients” [49]. In contrast, the effect of the common LFP seems to reside mainly in the low-frequency components of the signal (Figures 2, 5 and S1).

Regression analysis assumes that regressor variables are uncorrelated [42]. When using samples from an electrode array as regressors, both the noise and the signal are expected to be correlated spatially with a regular structure. The polynomial model further contributes to the multicollinearity, which can *e.g.* be dealt with using ridge regression [42]. Similarly, the hidden layer of an artificial neural network used for solving a regression problem will also compress and decorrelate the signals from the input layer [47]. However, as long as we are not predicting outside the “regressor variable hull”, and the covariance matrix is not too close to singularity, correcting for multicollinearity is not necessary [42]. A related issue is temporal correlation in the residuals. Generalized least squares models (GLS) (*cf. LAPACK* subroutines *GGGLM [50]) allow the temporal correlation structure to be modeled [42]. The Cochrane-Orcutt method [51] can also be used to obtain a “pseudo-GLS” estimate by pre-whitening the data with a first order autoregressive model. If a neural network is used for non-linear regression, it can deal with temporal correlation by recurrently feeding the residuals back to its input layer or by using Cochrane-Orcutt estimation. In our estimation procedure, we did not implement time-series modeling because good enough LFP prediction was achieved without this complicating procedure. We were also more interested in producting a good LFP prediction rather than obtaining unbiased regression coefficients for statistical inference [42].

### Waveform estimation with high-pass filters

We have demonstrated that frequency-dependent linear filters substantially distort the waveform of the extracellular spike (Figure 2). Such distortions by filtering are well known from signal processing theory [4]–[6], but these effects appear not to be widely known or considered among neuroscientists. The distortions occur as a result of frequency overlap between the spike and the LFP, combined with waveform artifacts introduced by the filter itself, including “ringing” and “non-linear phase response”. Waveform distortion of the extracellular spike occurs already with 12.5 Hz high-pass filters, and gets worse with increasing filtering frequency. This suggests that only the lowest frequency components of the LFP, theta and delta, do not contribute to the waveform of extracellular spikes from pyramidal cells in CA1. When a commonly used cut-off frequencies such as 300 or 600 Hz are used, the high-pass filter will actually intersect the wavelet spectrum of a typical extracellular spike. The waveform is also clearly distorted, and high-pass filtering also produced a marked negative bias in the estimate of extracellular spike amplitude (Figures 2 and S1).

The high-pass filtered EC action potential in Figure 2 is narrower than the raw signal, and it has acquired a trailing “AHP-like” bump. In fact, while the latter half of the raw spike signal produced a negative peak in the LFP, of approximately 5 ms duration, the filtered spike was trailed by a positive wave in the LFP, of approximately 0.5 ms duration. Figure 1B and the blue trace in Figure 4B shows the reason for this distortion: the slower negative wave associated with the EC action potential (*i.e.* not the spike) has a duration of approximately 8 ms. Assuming this is a half-period of a sinusoid, it has a spectral frequency of about 60 Hz. Consequently a 600 Hz low-pass filter responds to this with a wave that is slightly delayed compared to the spike. When the filter is inverted to high-pass, this is subtracted from the broad-band signal, and gives the false impression of an “AHP-like” bump trailing the spike. The delay results from the causal nature of the filter.

It is important not to give these filter artifacts false biophysical interpretations. The time from the negative peak of the spike to the peak of a positive bump following the spike has sometimes been called the “spike-width” [1], [21], as if that late bump was interpreted as an extracellular correlate of the spike repolarization (*i.e.* current flowing out if the cell during repolarization might be expected to produce a positive transient in the LFP). However, in our recordings, the positive bump following the spike disappeared when we used our multiple regression method instead of filtering, indicating that the positive bump seen in the filtered traces was merely an artifact generated intrinsically in the filter, at least in these cases. This result does not prove that the late part of any apparently biphasic HP-filtered EC spike waveform is always purely a filter artifact (there are indeed good reasons to expect real bi- or triphasic spike waveforms under certain conditions), but our analysis indicates that such filtering artifacts occur and can be quite prominent, and that they can be eliminated by the multiple regression method. Another biophysically false interpretation of this artificial “bump” would be to equate it with an extracellular correlate of the fast AHP or “undershoot” that often follows neuronal spikes. An extracellular spike filtered this way might superficially resemble “an AP with an AHP” if viewed upside down and if the duration is not taken into account.

Taken together, these results suggest that, although high-pass filtered extracellular spike recordings are useful for determining spike-timing, they should generally not be used for characterizing the biophysical properties of the action potentials.

### Waveform estimation with spike-triggered average

Averaging the broad-band signal recovered the correct spike waveforms, albeit with a larger standard deviation compared to the results of our procedure (Figure 8A–8C and 8G–8I). This larger waveform variability is dependent on the filtering frequency. As the recording was made using AC-coupled amplifiers, the raw signal can be seen as a trace that is high-pass filtered at approximately 0.5 Hz. The variability in the filtered spike decays with increasing filtering frequency. Averaging also introduced a systematic error depending on the phase-modulation of the spikes to the LFP (Figure 8). This is in contrast to our procedure, which avoids such systematic errors because it models and subtracts the LFP.

It is also important to distinguish between retrieving the *average waveform* of the EC action potentials from a cell, and retrieving the waveform of *individual* APs. Retrieving the average waveform of the EC spikes is useful if the waveform can be considered stationary. But there are conditions where the AP waveform from a single cell actually changes over time, such as during a complex spike burst (*i.e.*, which is characterized by frequency-dependent spike broadening and spike amplitude depression [8], [52], [53]. Various modulatory changes and fluctuations in neuronal membrane potential or conductance may have similar effects on the waveform of individual spikes.

### LFP cancellation by differential amplification is insufficient

One of the simplest ways to cancel common noise is to use a differential amplifier [6] to subtract a confounding signal from the recording. However, LFP cancellation by simply subtracting the recorded LFP is generally not possible because of the spatial variability in the LFP and the extracellular AP.

The electric range of a “tetrode” made from 13 µm NiCr wire is at least 100 µm [25]. Similarly, Lin *et al.* [54] estimated the electric recording horizon of a multielectrode array used in neuroscience to be less than 200 µm away, whereas Buzsaki and Kandel [46] reported 400 µm electrical radius for these recording probes. The linear electrode arrays from NeuroNexus Inc. that we used had 50 µm spacing between the recording sites, and we observed spikes from the same cell on four consecutive electrodes (i.e. across a distance of 150 µm). Previous studies using tetrodes made from 17 µm PtIr wire [12], [13] found that a spacing of 200 µm was sufficient to avoid spikes on the reference electrode. However, in the rodent hippocampus, the LFP can be very different at locations that far apart. The spatial ranges of the LFP and extracellular APs therefore require a more sophisticated estimation procedure for LFP suppression than a simple subtraction.

### Conclusion

Traditional linear filters are often insufficient for separating extracellularly (EC) recorded action potential waveforms from LFP, because the frequency bands of the LFP and spikes are overlapping. Because the commonly used high-pass filtering of action potentials often causes severe distortion of the spike waveform, it may even lead to false biophysical interpretations.

The novel procedure presented here, however, can correctly extract the real shape of the spike waveform with minimal distortion. By using multi-site electrode arrays with known geometry, parallel data acquisition, and multiple regression, our method was able to predict the LFP on channels with spikes from recording sites void of spikes, and produced smaller standard deviation than a spike-triggered average. This opens the possibility of using detailed analysis of EC spike waveforms for studying biophysical properties of the recorded cells, a method that may be exploited routinely for extracellular unit recordings *in vivo*.

## Materials and Methods

### Animals

The mice (male *C57bl6*) used for this study were part of an *in vivo* electrophysiology study of potassium channel function [55].

The mice were housed in transparent polycarbonate cages in a temperature and humidity-controlled vivarium with *ad libitum* access to food and water. Lights were on between 6 AM and 6 PM, and the recording was carried out during the light period.

The mice were brought to the laboratory thirty minutes before induction of urethane anesthesia (1.2 g/kg ethyl carbamate *i.p.*, followed by 20 mg/kg ketamine *i.p* ten minutes later). The scalp and throat were shaved and a trigeminal nerve-block of bupivacaine (5 mg/ml) and epinephrine (5 µg/ml) was administered *s.c.* above the eyes. The depth of anesthesia was measured from the pinna and pedal reflexes. The mice were intubated by tracheotomy as previously described [55]. A skin incision was made along the sagittal midline of the scalp, and the periost was removed. A stainless steel jeweller's screw was secured to the scull and served as ground electrode. A craniotomy was made above the right hippocampus using a trepan drill.

All experimental procedures were approved by the section for comparative medicine at the Insitute of Basic Medical Sciences, University of Oslo, and by the Norwegian animal research authority. The procedures complied with national laws and European Communities Council Directive of 24 November 1986 (86/609/EEC) governing the use of animals in research.

### Data collection

A 16-site 50 µm spaced laminar electrode array (*NeuroNexus Inc.*, Ann Arbor, MI) was inserted above the right hippocampus (2.0 mm caudal, 1.5 mm lateral, and 0.8 mm ventral to bregma). The electrode array was slowly lowered through the neocortex and into the hippocampus in steps of 20 µm per minute, until extracellular spikes from the CA1 pyramidal layer and the dentate gyrus were observed.

Recordings were made single-ended with AC-coupled differential amplifiers (*Axona ltd.*, St. Albans, UK), amplified 3000 times, broad-band filtered 0.1–6.8 kHz, and digitized at 16 kHz with 16 bits per sample resolution.

The recording equipment consisted of four arrays of four ADCs driven by the same clock. The relative clock-speeds of the ADC arrays were measured in advance. The digital raw data from all 16 data channels was synchronized off-line by upsampling to 16 GHz (1000 times the sampling rate) using a finite impulse-response (FIR) least-squares interpolation filter, and subsequently resampling to synchrony. The spikes were detected and the peaks time-stamped using a order-2 Butterworth filter (600 Hz) with a threshold of −50 µV. The signals were interpolated from 16 to 96 kHz prior to timing the spikes. Digital raw data upsampled to 96 kHz was sampled in time windows of ±100 ms around each spike (even larger windows were sampled for Figure 5B). Spikes were isolated by using in-house 3D cluster-cutting software (S. Molden). Cluster boundaries (Figure 7C and D) were visualized by shrinking a bounding sphere towards the centroid until vertices coicided with 5th nearest-neighbor distances, obtained from searching a 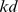-tree. Spikes on adjacent channels were considered to originate from the same AP on the heuristic criterions that (1) APs originate in the vicinity of the soma (50 µm is not sufficient to discern soma from initial axonal segment) and propagate away from soma, (2) that EC amplitude is largest in the vicinity of soma and decrease away from soma, and (3) that the speed of antidromic conduction was at least 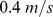. (Antidromic conduction speed of 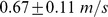 has been reported for neocortical pyramidal cells [46], we used 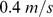 as a conservative lower limit since faster conduction will lead to even closer vicinity in time.)

All computations were performed with custom written Fortran 95 and Python software.

## Supporting Information

Figure S1 **High-pass filtering an average CA1 spike waveform.** The figure shows the effect of passing the spike waveform from Figure 1B through various high-pass filters, after inserting it into the recorded hippocampal local field potential. The red trace indicates the inserted waveform (and thus the desired output), the blue trace indicates the average of the filtered signal with standard deviation in the error bars. High-pass filtering frequencies increase from 12.5 Hz (top) to 800 Hz (bottom). An order-2 Butterworth filter is used for the left column, a single-pole (RC) filter is used for the right column. The filters are applied forwards and in reverse, and have zero delay. The filter-types were chosen because they correspond to common filter designs used for extracellular multi-unit recording. The gradual development of the waveform distortion is evident from the upper to the lower panels, with significant distortion already at 12.5 Hz for order-2 Butterworth and 12 Hz for RC. The waveform distortion includes narrowing of the spike, depression of the amplitude, and positive over-shoot after the spike. The red and blue arrows indicate the peak amplitude of the inserted signal (red traces) and the average filtered signals (blue traces). The distance between the blue and red arrow indicate the bias in the amplitude estimate. The development of the amplitude bias in the left column is also shown in greater detail by the dotted lines in Figure 5A–E.
(EPS)

Figure S2 **Waterfall plot of the spike-triggered hippocampal LFP.** The figure shows the spike triggered LFP of (**A**) the spike channel, (**B**) the closest reference channel, and (**C,D**) their averages. LFP at the time of subsequent spikes are stacked vertically. The spike is not visible on the closest reference channels.
(EPS)

Figure S3 **Effect of time-window on the regression estimate.** The figures shows the regression estimated with increasing time-windows, 15 ms to 960 ms. Particularly in the two upper panels, the error can be seen to increase with distance from the spike. This is due to inability to forecast (extrapolate) the LFP beyond the time-window used for fitting the model. This is mainly due to the use of a polynomial model and also multicollinearity in the regression variables, which interferes with using a regression model for extrapolation. However, it is of no significance for the longer time-windows (60 ms and larger).
(EPS)
